# Significant obesity‐associated gene expression changes occur in the stomach but not intestines in obese mice

**DOI:** 10.14814/phy2.12793

**Published:** 2016-05-20

**Authors:** Jing Chen, Lihong Chen, Philippe Sanseau, Johannes M. Freudenberg, Deepak K. Rajpal

**Affiliations:** ^1^ Computational BiologyTarget SciencesGlaxoSmithKlineKing of PrussiaPennsylvania; ^2^ Enteroendocrinology DPUGlaxoSmithKlineResearch Triangle ParkNorth Carolina

**Keywords:** Diet‐induced changes, metabolic diseases, obesity, obesity mechanisms, stomach, transcriptomics

## Abstract

The gastrointestinal (GI) tract can have significant impact on the regulation of the whole‐body metabolism and may contribute to the development of obesity and diabetes. To systemically elucidate the role of the GI tract in obesity, we performed a transcriptomic analysis in different parts of the GI tract of two obese mouse models: ob/ob and high‐fat diet (HFD) fed mice. Compared to their lean controls, significant changes in the gene expression were observed in both obese mouse groups in the stomach (ob/ob: 959; HFD: 542). In addition, these changes were quantitatively much higher than in the intestine. Despite the difference in genetic background, the two mouse models shared 296 similar gene expression changes in the stomach. Among those genes, some had known associations to obesity, diabetes, and insulin resistance. In addition, the gene expression profiles strongly suggested an increased gastric acid secretion in both obese mouse models, probably through an activation of the gastrin pathway. In conclusion, our data reveal a previously unknown dominant connection between the stomach and obesity in murine models extensively used in research.

## Introduction

The World Health Organization has warned about the new pandemic of obesity and its accompanying noncommunicable diseases (NCDs) such as diabetes with a projection of new cases of diabetes run into the hundreds of millions within the next 2 decades (World Health Organization, 2009). One of the potential causes of the pandemic is the change in diet composition and increased consumption of processed food products in recent decades. As part of the digestive system, the gastrointestinal tract (GI tract) is traditionally considered as a multiorgan system responsible for consuming and digesting foodstuffs, absorbing nutrients, and expelling waste. If dietary changes truly have a big impact on our health, the GI tract is exposed to those changes before the rest of the body. Although digestion is still the major function of the GI tract, it can also regulate the whole‐body metabolism via a combination of the complex enteric nervous system, enteroendocrine hormones, and tissue metabolism (Rubino et al. 2010). The importance of the GI tract in metabolic diseases is further supported by recent demonstration of significant improvement of glucose metabolism and decrease in body weight after certain bariatric surgeries (Rubino et al. 2010). Those benefits of bariatric surgeries cannot be explained by GI restriction and malabsorption and the effect on glucose is weight‐independent.

Unlike other traditional metabolic tissues such as the liver, muscle, and adipose, differences in the GI tract between normal and obese states have not been systemically explored at the transcriptional level to the best of our knowledge. In this study, we examined the gene expression in the GI tract of ob/ob and high‐fat diet (HFD) induced obese mice, two obese mouse models extensively used in research. The 6 parts of the GI tract examined in the study were stomach, duodenum, jejunum, ileum, ascending colon, and descending colon. The aims of this study were as follows: (1) to investigate which part of GI tract was significantly affected by obesity to identify potential GI contributions to the development of obesity; (2) to assess if significant differences between the two mouse models exist; and (3) to share the data generated during this study for the wider scientific community to potentially generate further knowledge.

## Materials and Methods

### Animals

All studies were conducted after being reviewed by the GlaxoSmithKline Institutional Animal Care and Use Committee and in accordance with the GlaxoSmithKline Policy on the Care, Welfare and Treatment on Laboratory Animals, The Animal Welfare Act (US Department of Agriculture), and the Guide for Care and Use of Laboratory Animals (Institute of Laboratory Animal resources, 1996).

For the first model, 9‐week‐old male, ob/ob (B6.V‐Lepob/J), and ob lean control (Lepob, heterozygote from the colony) mice were purchased from Jackson Lab (Bar Harbor, Main) and were acclimatized to a constant temperature and humidity (72°F and 50% relative humidity with a 12 h light and dark cycle from 0500 h to 1700 h) with free access to food (LabDiet 5K20, LabDiet, St. Louis, MO) and water. At the age of twelve weeks, all animals were fasted for4 h in the morning and euthanized at 1 pm under isoflurane anesthesia. Gastrointestinal tissues (whole stomach and about 1 cm samples from the middle of each part of the intestine) were collected and frozen immediately in liquid nitrogen and kept at −80°C for future RNA extraction.

For the second model, 9‐week‐old male, C57BL/6J mice were purchased from Jackson Lab (Bar Harbor, Main) and were acclimatized to a constant temperature and humidity (72°F and 50% relative humidity with a 12 h light and dark cycle from 0500 h to 1700 h) for 1 week with free access to food (LabDiet 5001, LabDiet, St. Louis, MO) and water. After acclimation, half of the mice (*n* = 6) were kept on the regular chow diet (LabDiet 5001) and half of them (*n* = 6) were switched for 4 weeks to TD93075 (Harlan Teklad, Madison, Wisconsin), a high‐fat diet with 54.8% kcal. At the end of the study, all animals were fasted for four hours in the morning and euthanized at 1 pm under isoflurane anesthesia. Tissues samples were collected as described above and frozen immediately on dry ice or in liquid nitrogen and kept at −80°C for future analyses.

### Sample preparation for microarray studies

RNA extraction was performed using the RNeasy Mini kit (Qiagen Inc, Valencia, Calif). Total RNA yield and purity was estimated by UV spectroscopy (Nanodrop ND‐1000 Spectrophotometer; Nanodrop Technologies, Wilmington, DE) and RNA quality was assessed on an Agilent 2100 Bioanalyzer. Subsequently mRNA expression analysis was carried out.

### Differential gene expression analysis

The gene expression profiles were derived from Affymetrics GeneChip Mouse Genome 430 2.0 Arrays. The microarray data were preprocessed, that is, background corrected and normalized, by Robust Multi‐array Average (RMA) (Irizarry et al. 2003) with custom chip definition file (CDF) downloaded from BrainArray (Dai et al. 2005). Data quality was assessed using Array Quality Metrics (Kauffmann et al. 2009). An unbiased filter was applied before differential expression analysis to remove the genes whose expression level was in the first quartile in all samples, that is, the genes that were constitutively not expressed in any samples.

The purpose of differential expression analysis was to identify genes that were differentially regulated in ob/ob compared to ob lean control in the first model, and HFD compared to normal diet in the second model in each separate GI tract tissue. The analysis was performed using Linear Models for Microarray and RNA‐Seq Data (limma) (Ritchie et al. 2015). Specifically, in each mouse model, each sample was characterized by three factors: (Amland et al. 1984) genotype or diet group of the sample (ob/ob or ob control in first model, or HFD or normal diet in the second model); (Arble et al. 2015) tissue type (stomach, duodenum, jejunum, ileum, ascending colon, or descending colon); and (Bandyopadhyay et al. 2015) the animal the sample was obtained from. Since multiple tissue samples were collected from each individual animal, the animal was considered as a random effect. Accordingly, a linear model with animal as random effect was fitted for each gene in each mouse model separately. The comparison between ob/ob versus ob control, or HFD versus normal diet was made for each tissue by computing the corresponding contrast. Genes with false discovery rate (FDR) adjusted *P*‐value <0.1 and absolute fold‐change >1.5 in the comparisons were selected as differentially regulated genes. Given FDR is relatively stringent, this cutoff was used for positive or negative calls in expression differentiation. However, if a gene had a significant *P*‐value and strong biological relevance but did not meet the cut‐off for FDR, we still included that gene in our hypotheses‐generating process. Those genes will have a p‐value listed in the text. By doing that, we tried to avoid missing important biological changes due to arbitrary cut‐off values. The entire gene‐level expression analysis and visualization were performed in the R statistical environment. The complete microarray data from this experiment were submitted to the GEO database with accession number GSE69306.

### Pathway analysis

The purpose of this analysis was to identify pathways that were overrepresented in up or downregulated genes comparing ob/ob versus ob control, or HFD versus normal diet in each tissue separately. All canonical pathways in MetaCore from Thomson Reuters were used for the pathway enrichment test, which estimated significance of each pathway based on a hyper geometric test. *P*‐value and the corresponding FDR adjusted p‐value were calculated. Pathways with FDR adjusted enrichment *P*‐value <0.1 were considered as significantly overrepresented pathways. The pathway enrichment analysis was performed using MetaBaseR Scripts Library 3.2.6 from Thomson Reuters under R statistical environment with MetaBase version 6.19.

## Results

Two independent experiments focusing on two different obese mouse models were performed. In this study, we profiled the transcriptomic changes in the GI tract tissues in a genetic‐induced and a diet‐induced obese mouse model, respectively. First, the results from the two mouse models were summarized separately, and then a combined analysis was performed to compare the two models.

### ob/ob model

#### Differentially expressed genes

**Figure 1:**
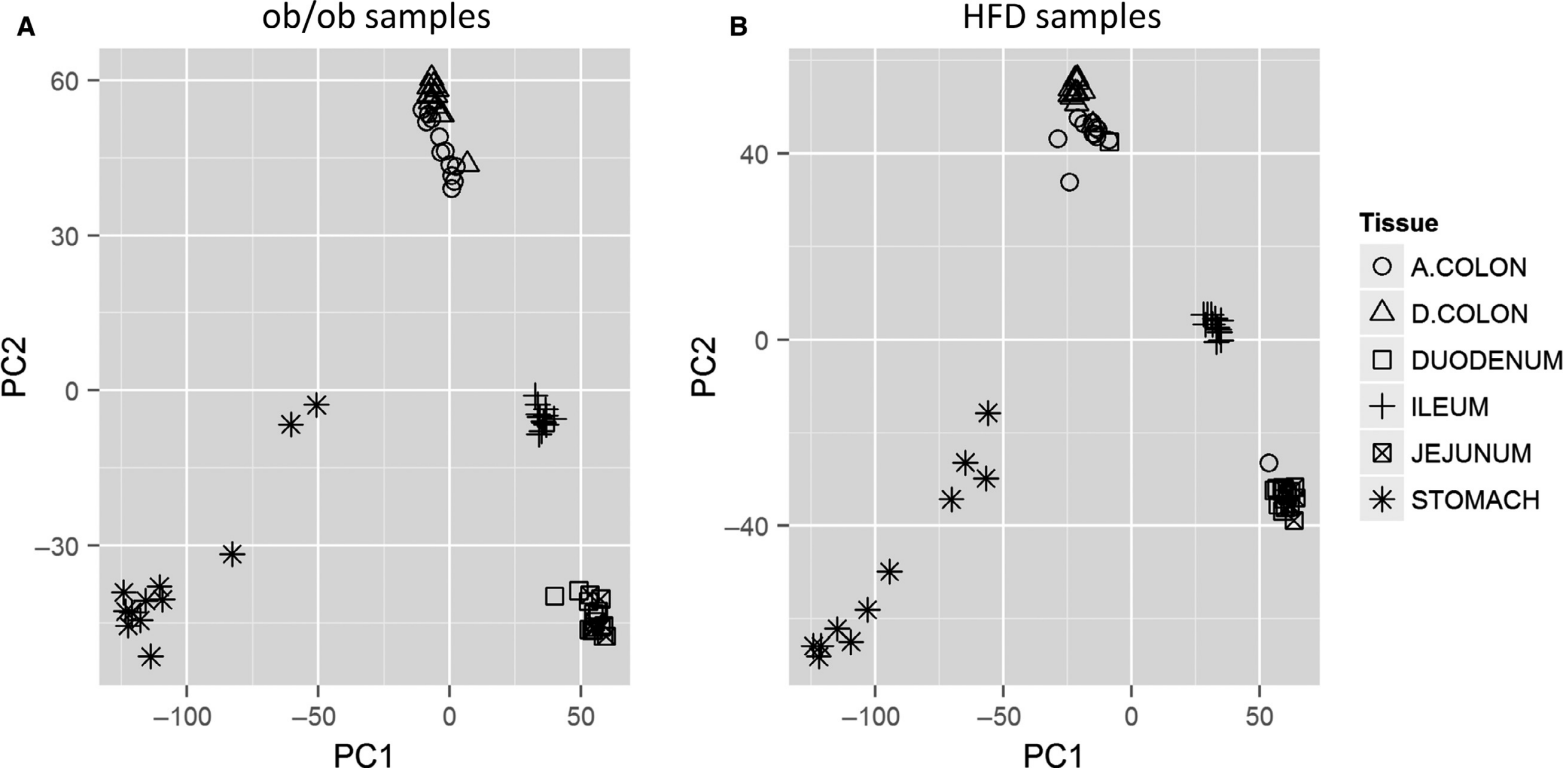
Plots of PCA result based on expression levels of all genes. (A) ob/ob and ob control samples. (B) HFD and normal diet samples. According to this plot, the samples are largely clustered by their tissue types, with ascending colon and descending colon clustered together, duodenum and jejunum clustered together, and stomach and ileum clustered separately.

There were two genotype groups in this experiment, ob/ob and ob control. There were six mice in each group, and samples from six GI tissues from each mouse were collected. Data from all microarray samples passed the standard Affymetrics quality metrics check and were used in the following analysis. A PCA plot (Fig. 1A) showed that the samples were clustered by their tissue types, with ascending colon and descending colon clustered together, duodenum and jejunum clustered together, and stomach and ileum clustered separately. This plot confirmed the quality of microarray data and suggested that the expression analysis should be performed for tissues separately.

**Figure 2:**
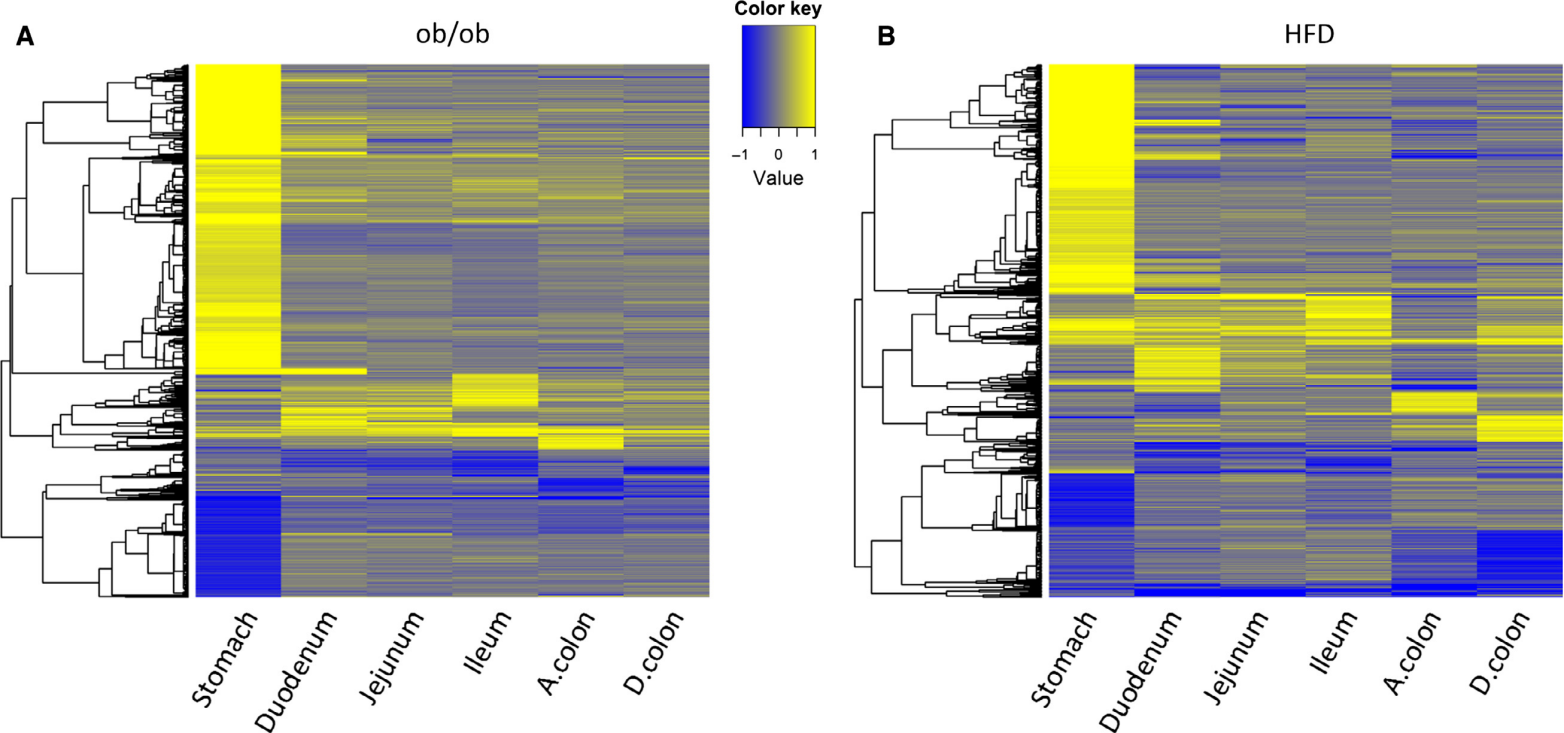
(A) Heat map of 1263 differentially expressed genes across different tissues, comparing ob/ob versus ob control samples. The log2 fold‐change profile between ob/ob versus ob control samples for each tissue is used for plotting. (B) Heat map of 926 differentially expressed genes across different tissues, comparing HFD versus normal diet samples. The log2 fold‐change profile between HFD versus control samples for each tissue was used for plotting. Yellow denotes up, and blue denotes downregulation in ob/ob or HFD. Genes and samples are represented by rows and columns, respectively. Genes with FDR < 0.1 and absolute fold‐change > 1.5 in any tissues are shown in the heat map and are clustered by hierarchical clustering.

The gene‐specific differential expression analysis was performed for each tissue, comparing ob/ob versus ob control with individual animal as a random effect. The numbers of up and downregulated genes in each tissue are summarized in Table 1. This table shows that the numbers of differentially regulated genes in ob/ob versus ob control varied across different tissue types, with many genes in stomach and very few in jejunum. A total of 1263 unique genes were differentially regulated in at least one tissue. A gene‐centric hierarchical clustering analysis of these genes across all tissue types were performed and the resulted heat map was plotted (Fig. 2A). Gene‐specific log2 fold‐change comparing ob/ob versus ob control for each tissue was used in the clustering analysis. As can be seen from the heat map, and consistent with Table 1, stomach showed not only the greatest number of differentially regulated genes, but also regulation patterns that were quite different from those in the other tissues.

**Table 1:** Number of significantly differentially regulated genes in ob/ob, HFD and both models, with FDR < 0.1 and absolute fold‐change > 1.5

Tissue	Up‐regulated			Down‐regulated			Opposite regulation in two models
	ob/ob	HFD	Common	ob/ob	HFD	Common	
Stomach	711	433	283	248	109	13	3
Duodenum	57	85	2	16	41	0	5
Jejunum	17	9	3	5	14	1	1
Ileum	103	76	7	51	36	2	11
A.colon	41	43	3	46	48	2	3
D.colon	17	60	4	30	95	2	1

Among the top 10 genes, sorted by absolute fold‐change of ob/ob versus ob control, nine were upregulated in ob/ob. The only downregulated gene was adipsin (Cfd), which was expressed 17‐fold less in stomach in ob/ob compared to ob control, and it was also downregulated in ileum and ascending colon. Another interesting observation was that many of these genes, such as Chia1, Gif, Pdia2, Anxa10, Atp4a, and Gsdma2, showed a stomach‐specific expression pattern. Their expression levels were at least 10 times higher in stomach than other GI tract tissues in the ob control samples. The complete analysis results for all genes in all tissues are available in the supplemental table.

#### Significant pathways

Pathways that were overrepresented in the up and downregulated genes in each tissue were identified separately through enrichment analysis. With redundant pathways removed, there were 20 unique pathways that were significantly overrepresented by upregulated genes (Table 2A) and 5 by downregulated genes (Table 2B) in at least one tissue. As can be seen from the table, many upregulated pathways were involved in metabolism, especially in stomach and ileum. On other hand, the downregulated pathways in stomach and descending colon were largely related to immune response.

**Table 2:** Over‐represented pathways in ob/ob mouse model. Numbers are counts of differentially regulated genes in each pathway in the specified tissue

Pathway	Stomach	Duodenum	Jejunum	Ileum	A.colon	D.colon
(A) Pathways over‐represented in up‐regulated genes						
Blood coagulation	15*	0	0	0	0	0
Bile acids regulation of glucose and lipid metabolism via FXR	10*	0	0	3#	0	0
Glycine, serine, cysteine and threonine metabolism	10*	0	0	0	0	0
Linoleic acid	6*	0	0	0	0	0
FXR‐dependent negative‐feedback regulation of bile acids concentration	6*	0	0	0	0	0
Platelet microparticle generation	14*	0	0	0	0	0
Phenylalanine metabolism	7*	0	0	0	0	0
PXR sigling via cross‐talk	6*	0	0	2#	0	0
Propiote metabolism p.1	5*	0	0	0	0	0
Leucine, isoleucine and valine metabolism.p.2	7*	0	0	0	0	0
Tight junctions	11*	0	0	0	0	0
(L)‐Alanine, (L)‐cysteine, and (L)‐methionine metabolism	6*	0	0	0	0	0
Sulfur metabolism	6*	0	0	0	0	0
FXR‐regulated cholesterol and bile acids cellular transport	6*	0	0	0	0	0
Triacylglycerol metabolism p.2	3#	3*	0	0	0	0
Glycolysis and gluconeogenesis p.1	0	0	0	4*	0	0
Fructose metabolism	5#	0	0	4*	0	0
GnRH sigling	0	0	0	6*	0	0
Aldosterone‐mediated regulation of EC sodium transport	0	0	0	2#	2#	3*
Impaired NO sigling in CF airways	0	0	0	0	0	2*
(B) Pathways over‐represented in down‐regulated genes						
Huntingtin‐depended transcription deregulation in Huntington's Disease	5*	0	0	0	0	0
Antigen presentation by MHC class II	4*	0	0	0	0	0
IFN alpha/beta sigling pathway	0	0	0	0	0	4*
Antiviral actions of Interferons	4#	0	0	0	0	4*
IFN gamma sigling pathway	0	0	0	0	0	3

*indicates FDR < 0.1 and ^#^ *P*‐value < 0.05 for the significance of the pathway in the specified tissue.

### HFD model

#### Differentially expressed genes

There were two diet groups in this experiment, HFD and normal diet. Six mice were used in each group, and samples from six tissues of each mouse were collected. Data from all microarray samples passed the standard Affymetrix quality metrics check and were used in the following analysis. The PCA plot (Fig. 1B) looked similar to the PCA plot in the ob/ob experiment. Four main clusters were observed in the plot: cluster of ascending and descending colon samples, cluster of duodenum and jejunum, cluster of stomach, and cluster of ileum samples.

The gene‐specific differential expression analysis was performed for each tissue, comparing HFD versus normal diet with individual animal as a random effect. The numbers of up and downregulated and unchanged genes in each tissue are summarized in Table 1. Just as the ob/ob model, the stomach was the tissue with the most differentially regulated genes in the diet‐induced model. In total, 926 unique genes were differentially regulated in at least one tissue. A gene‐centric hierarchical clustering analysis of these 926 genes was performed across all tissues and the heat map was plotted (Fig. 2B). Gene‐specific average log2 fold‐change comparing HFD versus normal diet for each tissue was used in the clustering analysis. As can be seen from the heat map, and consistent with Table 1, stomach showed not only the most differentially regulated genes, but also a different regulation pattern compared to other tissues. On other hand, some genes showed consistent regulation patterns in duodenum, jejunum, and ileum.

When sorted by absolute fold‐change of HFD versus normal diet, many of the top genes, for example, Chil4, Bpifb1, Myh1, Pdia2, Gif, Tnnc2, and Ltf, displayed a stomach‐specific differential regulation pattern. They had very low expression levels in intestinal tissues in both diet groups. However, their expression levels increased at least 12‐fold in stomach in the HFD group.

From the heat map in Figure 2B, it was observed that there was a group of genes that were consistently differentially regulated in all small intestine segments. By filtering the complete results of the gene‐level differential expression analysis, five genes (Tnfrsf17, Cyp1a1, Cyp2c55, Gstm1, and Trim13) were identified that were consistently and significantly differentially regulated in all the three segments of small intestine. Tnfrsf17 was upregulated by 1.8‐, 2.9‐, and 1.8‐fold in duodenum, jejunum, and ileum, respectively. Cyp1a1 was downregulated by 8.3‐, 10.7‐, and 3.1‐fold, and Cyp2c55 was downregulated by 46.0‐, 41.2‐, and 48.1‐fold in small intestines. Gstm1 expression levels were 7.0‐, 7.9‐, and 1.6‐fold lower in the three small intestine segments in the HFD group.

#### Significant pathways

Pathways that were overrepresented by the up and downregulated genes in each tissue were identified separately through enrichment analysis. With redundant pathways removed, there were 40 unique pathways that were significantly overrepresented by upregulated genes (Table 3A) and 30 by downregulated genes (Table 3B) in at least one tissue. Consistent with gene‐level result, stomach seemed to have a different set of overrepresented pathways than other GI tract tissues. For example, leucine, isoleucine, and valine (BCAA) metabolism pathway was associated with upregulated genes in stomach, whereas many immune response pathways were associated with upregulated genes in ileum. On other hand, pathways involved in cell cycle regulation, especially in metaphase checkpoint, were significantly overrepresented by downregulated genes in colon.

**Table 3:** Over‐represented pathways in HFD mouse model. Numbers are counts of differentially regulated genes in each pathway in the specified tissue

Pathway	Stomach	Duodenum	Jejunum	Ileum	A.colon	D.colon
(A) Pathways over‐represented in up‐regulated genes						
Leucine, isoleucine and valine metabolism.p.2	8*	0	0	0	0	0
Propiote metabolism p.1	5*	0	0	0	0	0
Tight junctions	9*	0	0	0	0	0
Blood coagulation	6*	0	0	0	0	0
(L)‐Alanine, (L)‐cysteine, and (L)‐methionine metabolism	5*	0	0	0	0	0
Populations of skin dendritic cells involved in contact hypersensitivity	0	0	0	5*	0	0
Role of B cells in SLE	0	2#	0	7*	0	0
Antiviral actions of Interferons	6#	0	0	5*	0	0
Th17 cell differentiation	0	0	0	5*	0	0
IL‐22 sigling pathway	0	0	0	5*	0	0
NK cells in allergic contact dermatitis	0	0	0	5*	0	0
Th17 cells in CF (mouse model)	0	0	0	5*	0	0
Differences between Langerhans cells and dermal dendritic cells in allergic	0	0	0	4*	0	0
Th17, Th22 and Th9 cell differentiation	0	0	0	5*	0	2#
ive CD4^+^ T cell differentiation	0	0	0	5*	0	2#
Th1 and Th2 cell differentiation	0	0	0	5*	0	0
Role of HMGB1 in dendritic cell maturation and migration	0	0	0	4*	0	2#
Th17 cells in CF	0	0	0	5*	0	0
Regulatory T cells in human allergic contact dermatitis	0	0	0	3*	0	0
LRRK2 and immune function in Parkinson's disease	0	0	0	4*	0	0
Mechanisms of hapten presentation to T cells in allergic contact dermatitis	0	0	0	3*	0	0
Differentiation of tural regulatory T cells	0	0	0	4*	0	0
NF‐AT sigling and leukocyte interactions	0	0	0	5*	0	0
Antigen presentation by MHC class II	0	0	0	3*	0	0
Differentiation and clol expansion of CD8^+^ T cells	0	0	0	4*	0	0
SLE genetic markers in B cell specific pathways	0	3#	0	5*	0	0
Generation of memory CD4^+^ T cells	0	0	0	4*	0	2#
Role of IL‐17‐producing T cells in allergic contact dermatitis	0	0	0	3*	0	0
T regulatory cell‐mediated modulation of antigen‐presenting cell functions	0	0	0	4*	0	0
Role of cell adhesion in vaso‐occlusion in Sickle cell disease	5#	0	0	4*	0	0
CD8^+^ Tc1 cells in allergic contact dermatitis	0	0	0	3*	0	0
Regulatory T cells in murine model of contact hypersensitivity	0	0	0	3*	0	0
T cell receptor sigling pathway	0	0	0	4*	0	0
NFAT in immune response	0	0	0	4*	0	0
Role of keratinocytes and Langerhans cells in skin sensitization	0	0	0	3*	0	0
G‐protein sigling_N‐RAS regulation pathway	0	0	0	3*	0	0
Inflammatory mechanisms of pancreatic	0	0	0	4*	0	3#
HSP60 and HSP70/ TLR sigling pathway 0 0	0	0	0	4*	0	3#
C3a sigling	0	0	0	4*	0	0
Reproduction_GnRH sigling	0	0	0	0	0	6*
(B) Pathways over‐represented in down‐regulated genes						
Cholesterol biosynthesis	4*	0	0	0	0	2#
CAR‐mediated direct regulation of xenobiotic metabolizing enzymes	0	7*	0	0	2#	2#
Benzo[a]pyrene metabolism	0	5*	4*	2*	0	0
2‐Naphthylamine and 2‐Nitronaphtalene metabolism	0	4*	2*	0	0	0
Retinol metabolism	2#	5*	3*	3*	0	0
Glutathione metabolism	0	5*	2#	0	3#	0
Naphthalene metabolism	0	4*	3*	0	0	0
Acetaminophen metabolism	0	3*	0	0	0	0
Estradiol metabolism	0	3*	2*	0	0	0
Glutathione metabolism	0	4*	0	0	2#	0
Estradiol metabolism	0	3*	2*	0	0	0
Androstenedione and testosterone biosynthesis and metabolism p.1	0	3*	2*	0	0	0
Serotonin ‐ melatonin biosynthesis and metabolism	0	3*	0	0	0	0
PXR‐mediated direct regulation of xenobiotic metabolizing enzymes	0	3*	0	2#	0	0
Vitamin K metabolism	0	2*	0	0	0	0
1‐Naphthylamine and 1‐Nitronaphtalene metabolism	0	2*	0	0	0	0
Aryl hydrocarbon receptor signaling	0	3*	0	2#	3#	3#
Estrone metabolism	0	2*	0	0	0	0
Histamine metabolism	0	2*	0	0	0	0
Estrone metabolism	0	2*	0	0	0	0
The metaphase checkpoint	0	0	0	0	0	12*
Role of APC in cell cycle regulation	0	0	0	0	0	13*
Chromosome condensation in prometaphase	0	0	0	0	0	10*
Spindle assembly and chromosome separation	0	0	0	0	0	11*
Role of Nek in cell cycle regulation	0	0	0	0	0	7*
Initiation of mitosis	0	0	0	0	0	6*
Progesterone‐mediated oocyte maturation	0	0	0	0	0	6*
Sister chromatid cohesion	0	0	0	0	0	4*
Abnormalities in cell cycle in SCLC	0	0	0	0	0	4*
Nucleocytoplasmic transport of CDK/Cyclins	0	0	0	0	0	3*

*indicates FDR < 0.1 and ^#^ *P*‐value < 0.05 for the significance of the pathway in the specified tissue.

The most significant pathway overrepresented by upregulated genes in stomach was BCAA metabolism pathway. Eight out of 24 measured genes in this pathway were upregulated, resulting in a *P*‐value of 1.5 × 10^−6^. For example, Aldh6a1, Acadsb, Mcc1, Mcc2, Pcca, and Pccb were upregulated by 2.3‐, 2.1‐, 2.0‐, 1.5‐, 1.7‐, and 1.6‐fold, respectively, in stomach in the HFD group.

The metaphase checkpoint in cell cycle was the most significantly overrepresented pathway in downregulated genes in descending colon in the HFD group. Twelve of 34 measured genes were downregulated in descending colon, which resulted in an enrichment *P*‐value of 7.3 × 10^−16^. For example, Cdc20, Aurka, Bub1, Mad2l1, Aurkb, and Bub1b were downregulated by 2.1‐, 1.9‐, 1.7‐, 1.7‐, 1.6‐, and 1.6‐fold, respectively, in descending colon.

### Combined analysis of ob/ob and HFD models

#### Comparison of the expression profiles between the two models

In the previous sections, the analysis of the two obese mouse models was performed separately. Here, we combine the two models and compare their transcriptomic changes. Since the two models were based on different genetics backgrounds and tested at different ages, direct comparisons of expression values were avoided. Instead, the normalized expression profiles relative to the corresponding controls were used, that is,. the gene‐level fold‐change of ob/ob versus ob lean control in the genetic model, and the fold‐change of HFD versus normal diet in the diet‐induced model. Spearman's correlation coefficients were first calculated between normalized expression profiles of any two tissues across the two models (Table 4). Surprisingly, stomach was the only tissue that showed significant concordance between the two models.

**Table 4:** Spearman's correlation coefficients of normalized expression profiles between all tissues of the two mouse models

	Hfd Stomach	Hfd Duodenum	Hfd Jejunum	Hfd Ileum	Hfd A.colon	Hfd D.colon
Ob Stomach	0.34	0	−0.12	−0.04	−0.22	−0.1
Ob Duodenum	0	−0.14	−0.06	−0.16	−0.17	−0.23
Ob Jejunum	0.04	−0.01	−0.05	−0.03	−0.18	−0.03
Ob Ileum	0.03	0.05	0.08	0.01	−0.08	0.03
Ob A.colon	0.05	0	0	0.05	−0.11	−0.06
Ob D.colon	−0.05	−0.08	−0.11	0	−0.23	−0.08

Second, the genes that were consistently up and downregulated in both models were identified for each tissue separately. The counts of these genes are listed in Table 1. The overlap between the differentially regulated genes in stomachs of the two models appeared a lot more significant than other tissues. For example, the numbers of upregulated genes were 711 and 433 in the two models, respectively, and the overlap was 283 genes (Fisher's exact *P*‐value < 1e‐100).

**Figure 3:**
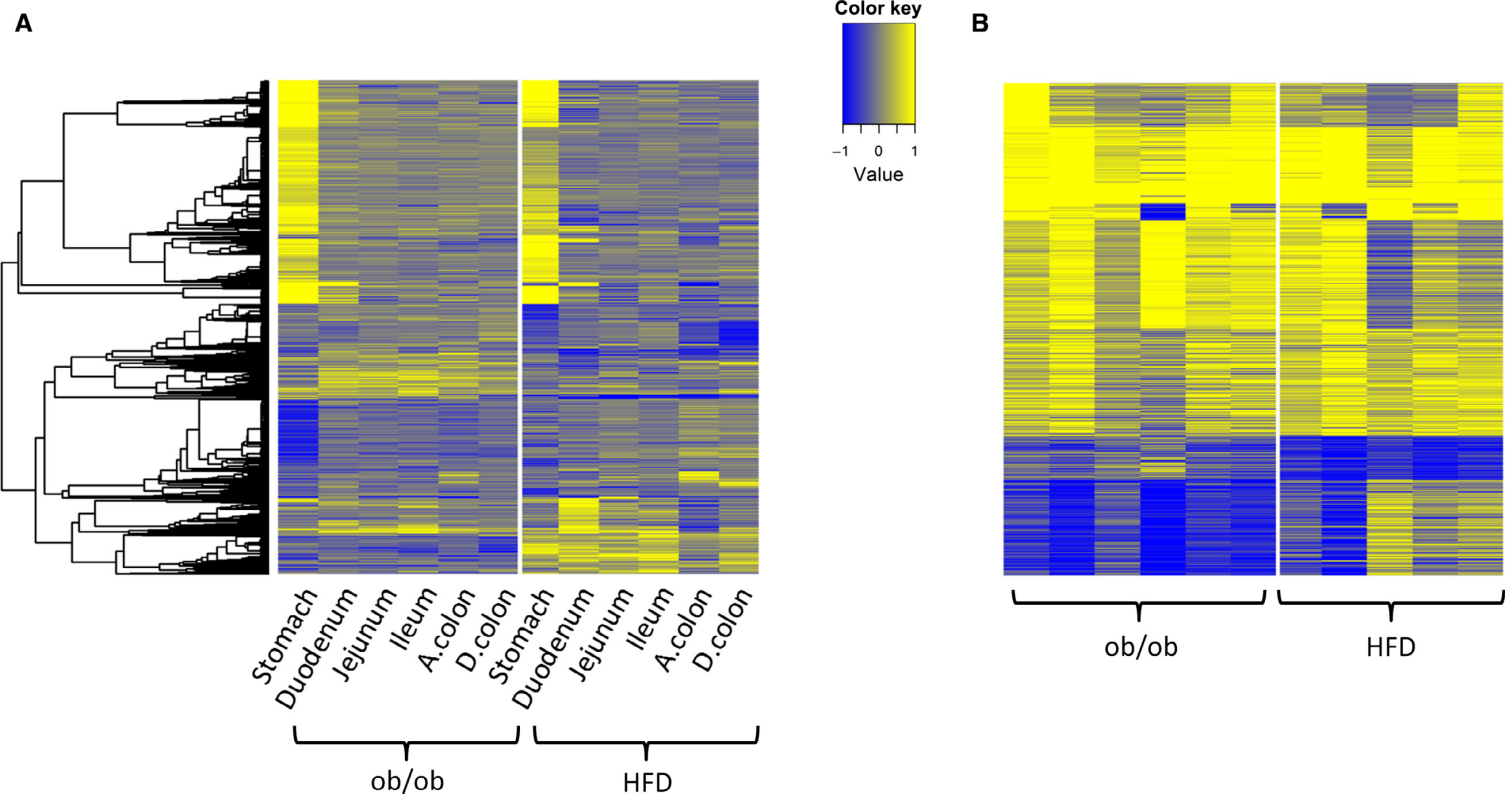
(A) Heat map of 1749 differentially expressed genes across different tissues in the two mouse models. The log2 fold‐change profile between ob versus ob control, or HFD versus normal diet is used. (B) Heat map of 1221 differentially expressed genes across different biological replicates of stomach in the two mouse models. The log2 fold‐change profile of each ob/ob sample normalized to ob control, or HFD sample normalized to normal diet is used. Yellow denotes up, and blue denotes downregulation in ob/ob. Genes and samples are represented by rows and columns, respectively. Genes with FDR < 0.1 and absolute fold‐change > 1.5 in any tissues are shown in the heat map and are clustered by hierarchical clustering.

Next, the genes that were differentially regulated in any tissue in either model were pooled together and a heat map of the expression profiles was plotted (Fig. 3A). In total, 1749 such genes were found and included in the heat map. According to the plot, and consistent with the results in previous sections, stomach showed the most significant gene changes in both models. Also noticeable and consistent with Table 1 results, many genes demonstrated similar expression changes between the two models in stomach, while there were very few such genes for other tissues.

Lastly, all the differentially regulated genes in stomach from the two models were pooled together. In total, 1221 such genes were found and a heat map of the normalized expression profiles was plotted (Fig. 3B). The normalized profile was calculated as the log2 fold‐change of each obese sample versus the average of corresponding lean controls. This plot clearly indicated, despite variances among biological replicates, that there were strong similarities in expression patterns for many genes between the stomachs of the two models.

#### Significant pathways of concordant genes in stomachs of the two models

In order to further characterize the genes that were commonly regulated in the two models, a pathway analysis was performed on the commonly up and downregulated genes. Eight pathways that were overrepresented in the 283 commonly upregulated genes in stomach were identified and are summarized in Table 5. No pathway was found that was overrepresented in the commonly downregulated genes in stomach. This was not surprising, since only 13 such genes were identified in the genome. In addition to the standard pathway analysis, we also performed investigations using alternative methods. Some examples, such as genes that are related to obesity (Table 6) and gastric acid secretion (Table 7), are discussed below in the Discussion section.

**Table 5:** Significant pathways of commonly up‐regulated genes in ob/ob and HFD

Pathway	Counts of up‐regulated genes
Tight junctions	9*
Blood coagulation	6*
Regulation of actin cytoskeleton by Rho GTPases	7*
Propionate metabolism p.1	4*
Stimulation of gastric acid secretion in gastric cancer	7*
Leucine, isoleucine and valine metabolism.p.2	5*
GPCRs in platelet aggregation	9*
Alpha‐1A adrenergic receptor‐dependent inhibition of	5*

*FDR < 0.1.

**Table 6:** Genes with known obesity association

GeneID	Gene symbol	Gene name	ob/ob	HFD
58991	Ghrl	Ghrelin	8.86*	14.35*
18778	Pla2g1b	Phospholipase A2, group IB, pancreas	9.32*	11.49*
22139	Ttr	Transthyretin	8.88*	8.44*
19662	Rbp4	Retinol binding protein 4, plasma	2.18*	1.90#
21784	Tff1	Trefoil factor 1	4.17*	2.97*
21785	Tff2	Trefoil factor 2 (spasmolytic protein 1)	7.24*	5.09*
69060	Pnlip	Pancreatic lipase	2.56	−1.56
18946	Pnliprp1	Pancreatic lipase related protein 1	9.34*	3.59*
18947	Pnliprp2	Pancreatic lipase‐related protein 2	9.84*	5.96*
20750	Spp1 (Opn)	Secreted phosphoprotein 1	6.47*	6.54*
12652	Chga	Chromogranin A	5.25*	4.79*

*FDR < 0.10, ^#^ *P* < 0.05.

**Table 7:** Gastric acid related genes

GeneID	Gene symbol	Gene name	ob/ob	HFD
11944	Atp4a	ATPase, H^+^/K^+^ exchanging, gastric, alpha polypeptide	10.11*	9.96*
11945	Atp4b	ATPase, H^+^/K^+^ exchanging, beta polypeptide	7.88*	10.74*
16535	Kcnq1	Potassium voltage‐gated channel, subfamily Q, member 1	3.92*	4.41*
246133	Kcne2	Potassium voltage‐gated channel, Isk‐related subfamily, gene 2	5.71*	9.82*
209195	Clic6	Chloride intracellular channel 6	7.52*	11.71*
20499	Slc12a7	Solute carrier family 12, member 7	2.08*	2.17*
20535	Slc4a2	Solute carrier family 4 (anion exchanger), member 2	2.35*	2.42*
208890	Slc26a7	Solute carrier family 26, member 7	1.44*	1.65*
226999	Slc9a2	Solute carrier family 9 (sodium/hydrogen exchanger), member 2	1.86*	1.96*
20544	Slc9a1	Solute carrier family 9 (sodium/hydrogen exchanger), member 1	1.58*	1.27
20496	Slc12a2	Solute carrier family 12, member 2	1.59*	1.32
11931	Atp1b1	ATPase, Na^+^/K^+^ transporting, beta 1 polypeptide	2.06*	2.20*
17829	Muc1	Mucin 1, transmembrane	5.72*	7.23*
17833	Muc5ac	Mucin 5, subtypes A and C, tracheobronchial/gastric	7.85*	3.66*
14459	Gast	Gastrin	1.17	−1.27
225642	Grp	Gastrin releasing peptide	−1.53*	−1.46
12426	Cckbr	Cholecystokinin B receptor	1.60*	1.31*
11829	Aqp4	Aquaporin 4	1.87	2.67#
11839	Areg	Amphiregulin	3.23*	1.94
15186	Hdc	Histidine decarboxylase	2.98*	2.13*
214084	Slc18a2	Solute carrier family 18 (vesicular monoamine), member 2	2.35*	3.22*
12652	Chga	Chromogranin A	5.25*	4.79*
12349	Car2	Carbonic anhydrase 2	1.53*	1.64
15466	Hrh2	Histamine receptor H2	1.65*	1.78*
14465	Gata6	GATA binding protein 6	2.38*	2.34*
20604	Sst	Somatostatin	4.61*	6.97*
20606	Sstr2	Somatostatin receptor 2	2.06*	1.39

*FDR < 0.10, ^#^ *P* < 0.05.

## Discussion

Although they vary in the magnitude of efficacy, most bariatric surgeries provide significant weight loss and both weight‐dependent and independent improvements in glucose metabolism and whole‐body insulin sensitivity (Maggard‐Gibbons et al. 2013). Some potential mechanisms have been proposed beyond restriction and malabsorption (Arble et al. 2015; Mingrone and Castagneto‐Gissey 2009; Rubino et al. 2006). Most if not all of those hypotheses focus on the intestine, but little is known about roles of the GI tract in the development of metabolic diseases such as obesity and diabetes, although some postprandial enteroendocrine responses have been reported (Madsbad 2014). To explore the contribution of the GI tract to obesity in a systemic way, we examined the GI gene expression in two of the most used obesity mouse models: HFD and ob/ob. The relative changes in gene expression were determined by comparing to age matched lean control groups. A 2‐week age difference between the two strains should not have a big impact on the interpretation of the results. Although body weights may be different, at 12–14 weeks of age, both strains have steady growth rates (www.jax.org). The gene expression results were unexpected in several ways. First, a significant number of differentially regulated genes were found in the stomach of both obese mouse models. Second, only a few gene expression changes were identified in different parts of the intestine. Third, while very few genes were regulated in the same direction in the intestine of the two obese mouse models, close to 300 genes were regulated in the same direction in the stomach of both ob/ob and HFD mice. Altogether, the data suggested that the stomach but not the other parts of the intestine might play a major role in the development of obesity. Because those gene expression changes were observed in both HFD and ob/ob mice, it was clear that the changes are not the result of a specific genetic background. More likely, those changes are associated with either a cause or a consequence of obesity. We further explored this possibility using two approaches: identifying known obesity‐associated genes and generating new hypotheses.

### Known obesity associations?

Most published gene expression analyses for obesity were done in traditional metabolic tissues such as adipose tissues or the liver. It is very interesting to notice that some of the differentially regulated genes in the stomach identified in this study have strong associations with obesity, diabetes, and insulin resistance in previous studies. Some examples include Ghrelin (Ghrl), a peptide produced by in the gastrointestinal tract that is also referred to as a “hunger hormone”. It functions as a neuropeptide in the central nervous system and stimulates food intake (Sato et al. 2012). The expression of ghrelin was significantly upregulated in the stomach of both obese mouse models. Group 1B phospholipase A (Arble et al. 2015) (Pla2g1b) expression was also significantly increased. It has been reported that mice lacking in Pla2g1b are resistant to obesity and diabetes induced by feeding a diabetogenic high‐fat/high‐carbohydrate diet (Huggins et al. 2002) and oral supplementation of a diabetogenic diet with the Pla2g1b inhibitor methyl indoxam effectively suppresses diet‐induced obesity and diabetes in mice (Hui et al. 2009). In addition, deficiency of Pla2g1b also significantly reduced atherosclerotic lesions in the aortic roots in Ldlr‐deficient mice (Hollie et al. 2014). Potential mechanisms may include the observation that lysophospholipids produced by Pla2g1b hydrolysis suppress hepatic fat utilization and downregulate energy expenditure (Labonte et al. 2010). Transthyretin (Ttr) is upregulated in both mouse models (*P *= 0.01 in HF). Circulating Ttr is a critical determinant of plasma retinol‐binding‐protein 4 (Rbp4) levels and lowering Ttr enhances the renal clearance of Rbp4. Elevated Rbp4 causes insulin resistance and lowering Rbp4 improves glucose homeostasis (Yang et al. 2005). Using Ttr antisense oligonucleotides (ASOs), Zemany and colleagues demonstrated that a reduction in circulating Ttr led to a decrease in Rbp4 in ob/ob and HFD mice with significant improvement in insulin sensitivity (Zemany et al. 2015). Interestingly, the expression of Rbp4 was also increased in the stomach of both models (*P *= 0.02 in HF). The expressions of trefoil factor 1 (Tff1) and trefoil factor 2 (Tff2, *P *= 0.01 in HF) were both increased in the stomach of ob/ob and HFD mice. Tff2 is a small gut peptide, mainly known for its protective and healing functions (Leung et al. 2002). De Giorgio and colleagues demonstrated that Tff2 might also be an important regulator in energy expenditure (De Giorgio et al. 2013). Tff2 deficient mice were protected from high‐fat diet induced obesity despite their greater appetite and higher energy intake compared to wild‐type animals. The expression of two paralogs of pancreatic lipase (Pnlip), Pnliprp1 (*P *= 0.03 in HF) and Pnliprp2 but not Pnlip are upregulated in the stomach of both ob/ob and HFD mice. All three genes are located on mouse chromosome 19 and clustered in two type 2 diabetes quantitative trait loci (QTL), Tanidd1, and T2dm2 (Brown et al. 2005). Pnliprp1 has undetectable lipase activity and may function as an inhibitor of dietary triglyceride digestion. Mice with inactive Pnliprp1 have high body fat composition without significant changes in body weight (Ren et al. 2011). They display impaired glucose tolerance and decreased insulin sensitivity, and obesity and insulin resistance are exacerbated by high‐fat diet. Their pancreatic juice has greater ability to hydrolyze triglycerides than that from wild‐type littermates. The expression of secreted phosphoprotein 1 (SPP1), also known as Osteopontin (OPN), was strongly induced in both models. In addition to its functions in biomineralization, bone remodeling, and immune response, osteopontin mediates obesity‐induced adipose tissue macrophage infiltration and insulin resistance in mice (Nomiyama et al. 2007), and neutralization of Osteopontin inhibits obesity‐induced inflammation and insulin resistance (Kiefer et al. 2010). Osteopontin deletion prevents the development of obesity and hepatic steatosis in mice (Lancha et al. 2014). Chromogranin A (Chga) is another gene whose expression is significantly increased in the stomach of both mouse models. Chromogranin A knockout (Chga‐KO) mice exhibit enhanced insulin sensitivity despite being obese (Bandyopadhyay et al. 2015). Changes in energy status associated with obesity and fasting, have been reported to alter mammalian target of rapamycin (mTOR) and nesfatin‐1, a satiety hormone (Li et al. 2012). In our study, mTOR was slightly upregulated in both models (1.28 and 1.22 for ob/ob and HFD), respectively. While a number of genes have been reported to be linked to obesity, we have not attempted to list them all, and rather show the significantly changed ones in tables. Because most of these genes encode (or generate) circulating factors, the current data may suggest that the stomach can play an important regulatory role in affecting whole‐body metabolism and the development of obesity, diabetes, and insulin resistance. Meanwhile, the above list is by no means exhaustive due to our analysis criteria and continuous increasing knowledge about obesity‐related genes.

### Gastric acid secretion

Additional analyses, including pathway analysis, suggested that some gastric functions might be altered in the stomach of both mouse models. Those changes could be pathophysiological causes or consequences of obesity and insulin resistance. Here, we focus on one novel hypothesis: gastric acid secretion.

By forming gastric acid no other tissue in the body builds higher concentration gradients for H^+^ (1‐million fold enrichment) than stomach mucosa. Gastric acid affects the digestive system in many ways including a key role in the digestion of proteins by activating digestive enzymes. Acid is secreted by parietal cells in the stomach via a coordination of multiple biochemical pathways and the whole process is regulated by multiple feedback mechanisms (Waldum et al. 1991). The luminal H^+^‐K^+^‐ ATPase drives gastric acid secretion (DuBose et al. 1999), but it is unable to pump H^+^ into the lumen of stomach without parallel uptake of K^+^. Meanwhile, the exit of K^+^ through K^+^ channels is countered (or activated) by luminal exit of Cl^−^ channels (Heitzmann and Warth 2007; Shin et al. 2009). In this study, both alpha and beta subunits of the H^+^‐K^+^‐ATPase (Atp4a and Atp4b) were dramatically upregulated in the stomach of both obese mouse models. Accompanying those changes, mRNA levels of several K^+^ channels were significantly increased. These included a key luminal K^+^ channel, Kcnq1, and its subunit Kcne2 (Heitzmann and Warth 2007). It has been proposed that Clic6 is the necessary accompanying Cl channel for gastric acid secretion (Shin et al. 2009). The expression of Clic6 was also significantly upregulated in this study. In addition, Slc12a7, a K^+^‐Cl^−^ cotransporter functionally associated with H^+^‐K^+^‐ATPase was also upregulated (Fujii et al. 2009). Some transporters on the basolateral side of parietal cells are important for maintaining intracellular pH homeostasis, volume regulation, and survival of parietal cells (Heitzmann and Warth 2007). Many of them including Slc4a2, Slc26a7, and Slc9a2 were upregulated. Slc12a2 and Slc9a1 expression was also increased in ob/ob but not HFD mice. Altogether, the gene expression profile strongly suggested an enhanced gastric acid secretion in ob/ob and HFD mice compared to their lean controls. Meanwhile, stomach also has a unique system to prevent the potential damage of gastric acid to the mucosa. The mucosa is always covered by a layer of thick mucus that is secreted by tall columnar epithelial cells. The expression levels of two major mucins of stomach, Muc1 and Muc5ac (Lau et al. 2004), were both increased in the ob/ob and HFD mice. While we speculate increased secretion of mucins as indirect supporting evidence for enhanced gastric acid secretion, further studies should be carried out to demonstrate the increased expression through immunohistochemistry and/or histology work.

Gastric acid secretion can be regulated in both direct and indirect ways by several hormones and neurotransmitters including gastrin and histamine (Schubert 1999). Gastrin, released from endocrine G cells in the distal portion of the stomach, is the principal hormone regulating gastric acid secretion (Schubert 1999). Neither gastrin nor gastrin‐releasing peptide (a neuropeptide that stimulates the release of gastrin) was changed at the transcriptional level in this study. However, the expression of the gastrin receptor (Cckbr) was significantly increased in ob/ob mice. Although not reaching the 1.5‐fold cut‐off (1.31‐fold), the difference of Cckbr mRNA levels between the HFD and normal mice was statistically significant. It has been demonstrated that gastrin can induce the expression of gastric acid secretion‐related genes such as H^+^‐K^+^‐ATPase *α*‐ and *β*‐subunits and Kcnq1 (Jain et al. 2006). In addition, we explored further whether the gastrin pathway was activated or not in obese mice by investigating genes whose expression could be increased by gastrin, namely: Aqp4, Areg, Hdc, Slc18a2, Chga, and Car2 (Jain et al. 2006). There was a trend for an increased expression of these genes if they were not significantly upregulated in both obese mouse models. Gastrin, by increasing the expression of Hdc, Slc18a2 and Chga in enterochromaffin‐like (ECL) cells, can stimulate the release of histamine (Jain et al. 2006). Histamine is another strong secretagogue for gastric acid. Histamine binds to histamine receptor H2 (Hrh2) and increases cAMP levels in parietal cells. Hrh2 expression was increased in this study. Gate6 (increased in both models) could be a key transcriptional factor for gastrin‐mediated transcriptional effects via MAP kinase/PI3 kinase pathways and Cdx (Dimaline et al. 1997; Leung‐Theung‐Long et al. 2005; Sun‐Wada et al. 2004). An increase in gastric acid secretion was further supported by the upregulation of a negative feedback system in the stomach. D cells in the antrum of stomach can sense low pH and increase the production and release of somatostatin (Sst), a hormone that will inhibit gastric acid secretion through somatostatin receptor 2 (Sstr2) (Schubert 1999). Sst expression was significantly increased in both obese mouse models. Sstr2 was significantly increased in ob/ob mice and had a trend to be higher in HFD mice compared to lean controls. Histological assessment of similar studies, may further reveal whether there are changes in the number of D cells, because an increased production of somatostatin may or may not be associated with a higher number of D cells. Altogether, the current gene expression profile strongly suggests that an overactivated gastrin pathway enhance gastric acid secretion in ob/ob and HFD mice.

### Relevance to obesity

Since our interpretation of the data is solely based on gene expression at the transcriptional level, we do recognize that the lack of functional data is a limitation of this study. In addition, we did not explore potential gender differences in this study. However, as outlined above, we believe that the data strongly suggest a connection between the stomach and the development of obesity. This conclusion is also supported by bariatric surgeries, most of which have some impacts on gastric function including gastric acid secretion (Melissas et al. 2002). In fact, using different surgery procedures, Patel and colleagues revealed a dominant role of the stomach in the regulation of body weight and incretin response to oral glucose (Patel et al. 2014). However, the exact role of gastric acid in obesity is not clear. A discrepancy between studies, including ours, may not only be caused by different assay conditions but also reflects the complexity of physiology. Some of them can only be addressed in specifically and properly designed studies. In animal models, hypergastrinemia is associated with the development of obesity (Morton et al. 1985; Pederson et al. 1989). Gastrin and gastric acid measurements in obese subjects produced variable results in different studies (Amland et al. 1984; Sasaki et al. 1983; Wisen et al. 1987; Zwirska‐Korczala et al. 2007). While showing no difference on circulating gastrin levels, Wisen and colleagues demonstrated that obese patients had increased sensitivity to pentagastrin (Wisen et al. 1987). Inhibiting gastric acid secretion using histamine H2 receptor antagonist or H^+^‐K^+^‐ATPase inhibitor also provided mixed results on body weight in human clinical studies (Birketvedt et al. 2000; Stoa‐Birketvedt et al. 1998; Yoshikawa et al. 2009). It is important to notice that some of the agents may also act on other targets in the GI tract (Nies et al. 2011). Our data demonstrated an increase expression of somatostatin perhaps indicating increased production from D cells. Although somatostatin analogs were proposed as a treatment for obesity, somatostatin actually reduced postprandial sensations after a satiating meal in obese individuals (Cremonini et al. 2005). Vaccines that induced high levels of antisomatostatin antibodies significantly reduced weight gain in HFD mice (Haffer 2012). Therefore, the increased somatostatin production may be the key connection between gastric acid secretion and obesity but more animal and human studies are needed to verify this hypothesis.

In summary, we demonstrate for the first time that obesity is associated with significant gene expression changes in the stomach but not in the rest of the GI tract. Some of these genes encode proteins (e.g., circulating factors) that have connections to obesity. The gene expression profiles strongly suggest an increased gastric acid secretion in obese mice, which can potentially be associated with the development of obesity. It is important to note that, genes mentioned above only form a small fraction of all genes changed in the stomach of both obese mouse models. The current data provide an opportunity to further explore the role of stomach in metabolic diseases.

## Conflict of Interest

None declared.

## Supporting information




**Table S1.** obese_mouse_models_complete_gene_results.xlsx: The complete analysis results for all genes in all tissues of both obese mouse models.

## References

[phy212793-bib-0001] Amland, P. F. , R. Jorde , K. E. Giercksky , S. Kildebo , and P. G. Burhol . 1984 Fasting and postprandial serum gastrin levels in obese and slim subjects, and the effects of surgical treatment for obesity on serum gastrin levels. Acta Chir. Scand 150:223–228. 6464625

[phy212793-bib-0002] Arble, D. M. , D. A. Sandoval , and R. J. Seeley . 2015 Mechanisms underlying weight loss and metabolic improvements in rodent models of bariatric surgery. Diabetologia 58:211–220. 25374275 10.1007/s00125-014-3433-3 PMC4289431

[phy212793-bib-0003] Bandyopadhyay, G. K. , M. Lu , E. Avolio , J. A. Siddiqui , J. R. Gayen , J. Wollam , et al. 2015 Pancreastatin‐dependent inflammatory signaling mediates obesity‐induced insulin resistance. Diabetes 64:104–116. 25048197 10.2337/db13-1747

[phy212793-bib-0004] Birketvedt, G. S. , E. Thom , B. Bernersen , and J. Florholmen . 2000 Combination of diet, exercise and intermittent treatment of cimetidine on body weight and maintenance of weight loss. A 42 months follow‐up study. Med. Sci. Monit. 6:699–703. 11208394

[phy212793-bib-0005] Brown, A. C. , W. I. Olver , C. J. Donnelly , M. E. May , J. K. Naggert , D. J. Shaffer , et al. 2005 Searching QTL by gene expression: analysis of diabesity. BMC Genet. 6:12. 15760467 10.1186/1471-2156-6-12 PMC555939

[phy212793-bib-0006] Cremonini, F. , M. Camilleri , J. Gonenne , D. Stephens , L. Oenning , K. Baxter , et al. 2005 Effect of somatostatin analog on postprandial satiation in obesity. Obes. Res. 13:1572–1579. 16222060 10.1038/oby.2005.193

[phy212793-bib-0007] Dai, M. , P. Wang , A. D. Boyd , G. Kostov , B. Athey , E. G. Jones , et al. 2005 Evolving gene/transcript definitions significantly alter the interpretation of GeneChip data. Nucleic Acids Res. 33:e175. 16284200 10.1093/nar/gni179 PMC1283542

[phy212793-bib-0008] De Giorgio, M. R. , M. Yoshioka , I. Riedl , O. Moreault , R. G. Cherizol , A. A. Shah , et al. 2013 Trefoil factor family member 2 (Tff2) KO mice are protected from high‐fat diet‐induced obesity. Obesity (Silver Spring) 21:1389–1395. 23754443 10.1002/oby.20165

[phy212793-bib-0009] Dimaline, R. , B. J. Campbell , F. Watson , A. K. Sandvik , J. Struthers , and P. J. Noble . 1997 Regulated expression of GATA‐6 transcription factor in gastric endocrine cells. Gastroenterology 112:1559–1567. 9136834 10.1016/s0016-5085(97)70037-4

[phy212793-bib-0010] DuBose, T. D. J. , J. Gitomer , and J. Codina . 1999 H^+^, K^+^‐ATPase. Curr. Opin. Nephrol. Hypertens. 8:597–602. 10541223 10.1097/00041552-199909000-00011

[phy212793-bib-0011] Fujii, T. , Y. Takahashi , A. Ikari , M. Morii , Y. Tabuchi , K. Tsukada , et al. 2009 Functional association between K^+^‐Cl^−^ cotransporter‐4 and H^+^, K^+^‐ATPase in the apical canalicular membrane of gastric parietal cells. J. Biol. Chem. 284:619–629. 18984587 10.1074/jbc.M806562200

[phy212793-bib-0012] Haffer, K. 2012 Effects of novel vaccines on weight loss in diet‐induced‐obese (DIO) mice. J. Anim. Sci. Biotechnol. 3:21. 22958753 10.1186/2049-1891-3-21 PMC3436618

[phy212793-bib-0013] Heitzmann, D. , and R. Warth . 2007 No potassium, No acid: K^+^ channels and gastric acid secretion. Physiology 22:335–341. 17928547 10.1152/physiol.00016.2007

[phy212793-bib-0014] Hollie, N. I. , E. S. Konaniah , C. Goodin , and D. Y. Hui . 2014 Group 1B phospholipase A(2) inactivation suppresses atherosclerosis and metabolic diseases in LDL receptor‐deficient mice. Atherosclerosis 234:377–380. 24747111 10.1016/j.atherosclerosis.2014.03.027 PMC4037866

[phy212793-bib-0015] Huggins, K. W. , A. C. Boileau , and D. Y. Hui . 2002 Protection against diet‐induced obesity and obesity‐related insulin resistance in Group 1B PLA2‐deficient mice. Am. J. Physiol. Endocrinol. Metab. 283:E994–E1001. 12376327 10.1152/ajpendo.00110.2002

[phy212793-bib-0016] Hui, D. Y. , M. J. Cope , E. D. Labonte , H. T. Chang , J. Shao , E. Goka , et al. 2009 The phospholipase A(2) inhibitor methyl indoxam suppresses diet‐induced obesity and glucose intolerance in mice. Br. J. Pharmacol. 157:1263–1269. 19563529 10.1111/j.1476-5381.2009.00308.x PMC2743845

[phy212793-bib-0017] Irizarry, R. A. , B. Hobbs , F. Collin , Y. D. Beazer‐Barclay , K. J. Antonellis , U. Scherf , et al. 2003 Exploration, normalization, and summaries of high density oligonucleotide array probe level data. Biostatistics 4:249–264. 12925520 10.1093/biostatistics/4.2.249

[phy212793-bib-0018] Jain, R. N. , C. S. Brunkan , C. S. Chew , and L. C. Samuelson . 2006 Gene expression profiling of gastrin target genes in parietal cells. Physiol. Genomics 24:124–132. 16278279 10.1152/physiolgenomics.00133.2005

[phy212793-bib-0019] Kauffmann, A. , R. Gentleman , and W. Huber . 2009 arrayQualityMetrics–a bioconductor package for quality assessment of microarray data. Bioinformatics 25:415–416. 19106121 10.1093/bioinformatics/btn647 PMC2639074

[phy212793-bib-0020] Kiefer, F. W. , M. Zeyda , K. Gollinger , B. Pfau , A. Neuhofer , T. Weichhart , et al. 2010 Neutralization of osteopontin inhibits obesity‐induced inflammation and insulin resistance. Diabetes 59:935–946. 20107108 10.2337/db09-0404 PMC2844841

[phy212793-bib-0021] Labonte, E. D. , P. T. Pfluger , J. G. Cash , D. G. Kuhel , J. C. Roja , D. P. Magness , et al. 2010 Postprandial lysophospholipid suppresses hepatic fatty acid oxidation: the molecular link between group 1B phospholipase A2 and diet‐induced obesity. FASEB J. 24:2516–2524. 20215528 10.1096/fj.09-144436 PMC2887262

[phy212793-bib-0022] Lancha, A. , A. Rodriguez , V. Catalan , S. Becerril , N. Sainz , B. Ramirez , et al. 2014 Osteopontin deletion prevents the development of obesity and hepatic steatosis via impaired adipose tissue matrix remodeling and reduced inflammation and fibrosis in adipose tissue and liver in mice. PLoS ONE 9:e98398. 24871103 10.1371/journal.pone.0098398 PMC4037189

[phy212793-bib-0023] Lau, S. K. , L. M. Weiss , and P. G. Chu . 2004 Differential expression of MUC1, MUC2, and MUC5AC in carcinomas of various sites: an immunohistochemical study. Am. J. Clin. Pathol. 122:61–69. 15272531 10.1309/9R66-73QE-C06D-86Y4

[phy212793-bib-0024] Leung, W. K. , J. Yu , F. K. Chan , K. F. To , M. W. Chan , M. P. Ebert , et al. 2002 Expression of trefoil peptides (TFF1, TFF2, and TFF3) in gastric carcinomas, intestinal metaplasia, and non‐neoplastic gastric tissues. J. Pathol. 197:582–588. 12210076 10.1002/path.1147

[phy212793-bib-0025] Leung‐Theung‐Long, S. P. , E. Roulet , P. Clerc , C. Escrieut , S. Marchal‐Victorion , B. Ritz‐Laser , et al. 2005 Essential interaction of Egr‐1 at an islet specific response element for basal and gastrin‐dependent glucagon gene transactivation in pancreatic alpha cells. J. Biol. Chem. 280:7976–7984. 15611055 10.1074/jbc.M407485200

[phy212793-bib-0026] Li, Z. , G. Xu , Y. Li , J. Zhao , M. W. Mulholland , and W. Zhang . 2012 mTOR‐dependent modulation of gastric nesfatin‐1/NUCB2. Cell. Physiol. Biochem. 29:493–500. 22508056 10.1159/000338503 PMC3711577

[phy212793-bib-0027] Madsbad, S. 2014 The role of glucagon‐like peptide‐1 impairment in obesity and potential therapeutic implications. Diabetes Obes. Metab. 16:9–21. 23617798 10.1111/dom.12119

[phy212793-bib-0028] Maggard‐Gibbons, M. , M. Maglione , and M. Livhits . 2013 Bariatric surgery for weight loss and glycemic control in nonmorbidly obese adults with diabetes: a systematic review. JAMA 309:2250–2261. 23736734 10.1001/jama.2013.4851

[phy212793-bib-0029] Melissas, J. , E. Kampitakis , G. Schoretsanitis , J. Mouzas , E. Kouroumalis , and D. D. Tsiftsis . 2002 Does reduction in gastric acid secretion in bariatric surgery increase diet‐induced thermogenesis? Obes. Surg. 12:399–403. 12082896 10.1381/096089202321088246

[phy212793-bib-0030] Mingrone, G. , and L. Castagneto‐Gissey . 2009 Mechanisms of early improvement/resolution of type 2 diabetes after bariatric surgery. Diabetes Metab. 35:518–523. 20152737 10.1016/S1262-3636(09)73459-7

[phy212793-bib-0031] Morton, A. P. , P. J. Hanson , and G. J. Dockray . 1985 Elevated serum and antral gastrin in obese (ob/ob) mice. Gastroenterology 88:945–950. 3972234 10.1016/s0016-5085(85)80012-3

[phy212793-bib-0032] Nies, A. T. , U. Hofmann , C. Resch , E. Schaeffeler , M. Rius , and M. Schwab . 2011 Proton pump inhibitors inhibit metformin uptake by Organic Cation Transporters (OCTs). PLoS ONE 6:e22163. 21779389 10.1371/journal.pone.0022163 PMC3136501

[phy212793-bib-0033] Nomiyama, T. , D. Perez‐Tilve , D. Ogawa , F. Gizard , Y. Zhao , E. B. Heywood , et al. 2007 Osteopontin mediates obesity‐induced adipose tissue macrophage infiltration and insulin resistance in mice. J. Clin. Invest. 117:2877–2888. 17823662 10.1172/JCI31986 PMC1964510

[phy212793-bib-0034] Patel, R. T. , A. P. Shukla , S. M. Ahn , M. Moreira , and F. Rubino . 2014 Surgical control of obesity and diabetes: the role of intestinal vs. gastric mechanisms in the regulation of body weight and glucose homeostasis. Obesity 22:159–169. 23512969 10.1002/oby.20441

[phy212793-bib-0035] Pederson, R. A. , R. V. Campos , C. B. Chan , A. M. Buchan , M. B. Wheeler , and J. C. Brown . 1989 Gastrin release in obese Zucker rats. Regul. Pept. 24:131–142. 2564209 10.1016/0167-0115(89)90232-2

[phy212793-bib-0036] Ren, J. , Z. Chen , W. Zhang , L. Li , R. Sun , C. Deng , et al. 2011 Increased fat mass and insulin resistance in mice lacking pancreatic lipase‐related protein 1. J. Nutr. Biochem. 22:691–698. 21115337 10.1016/j.jnutbio.2010.06.002

[phy212793-bib-0037] Ritchie, M. E. , B. Phipson , D. Wu , Y. Hu , C. W. Law , W. Shi , et al. 2015 Limma powers differential expression analyses for RNA‐sequencing and microarray studies. Nucleic Acids Res. 43:e47. 25605792 10.1093/nar/gkv007 PMC4402510

[phy212793-bib-0038] Rubino, F. , A. Forgione , D. E. Cummings , M. Vix , D. Gnuli , G. Mingrone , et al. 2006 The mechanism of diabetes control after gastrointestinal bypass surgery reveals a role of the proximal small intestine in the pathophysiology of Type 2 diabetes. Ann. Surg. 244:741–749. 17060767 10.1097/01.sla.0000224726.61448.1b PMC1856597

[phy212793-bib-0039] Rubino, F. , S. L. R'bibo , F. del Genio , M. Mazumdar , and T. E. McGraw . 2010 Metabolic surgery: the role of the gastrointestinal tract in diabetes mellitus. Nat. Rev. Endocrinol. 6:102–109. 20098450 10.1038/nrendo.2009.268 PMC2999518

[phy212793-bib-0040] Sasaki, H. , M. Nagulesparan , A. Dubois , E. Straus , I. M. Samloff , W. H. Lawrence , et al. 1983 Hypergastrinemia in obese noninsulin‐dependent diabetes: a possible reflection of high prevalence of vagal dysfunction. J. Clin. Endocrinol. Metab. 56:744–750. 6833461 10.1210/jcem-56-4-744

[phy212793-bib-0041] Sato, T. , Y. Nakamura , Y. Shiimura , H. Ohgusu , K. Kangawa , and M. Kojima . 2012 Structure, regulation and function of ghrelin. J. Biochem. 151:119–128. 22041973 10.1093/jb/mvr134

[phy212793-bib-0042] Schubert, M. L. 1999 Regulation of gastric acid secretion. Curr. Opin. Gastroenterol. 15:457–462. 17023991 10.1097/00001574-199911000-00002

[phy212793-bib-0043] Shin, J. M. , K. Munson , O. Vagin , and G. Sachs . 2009 The gastric HK‐ATPase: structure, function, and inhibition. Pflugers Arch. 457:609–622. 18536934 10.1007/s00424-008-0495-4 PMC3079481

[phy212793-bib-0044] Stoa‐Birketvedt, G. , P. N. Paus , R. Ganss , O. C. Ingebretsen , and J. Florholmen . 1998 Cimetidine reduces weight and improves metabolic control in overweight patients with type 2 diabetes. Int. J. Obes. Relat. Metab. Disord. 22:1041–1045. 9822940 10.1038/sj.ijo.0800721

[phy212793-bib-0045] Sun‐Wada, G. H. , Y. Kamei , Y. Wada , and M. Futai . 2004 Regulatory elements directing gut expression of the GATA 6 gene during mouse early development. J. Biochem. 135:165–169. 15047717 10.1093/jb/mvh019

[phy212793-bib-0046] Waldum, H. L. , A. K. Sandvik , E. Brenna , and H. Petersen . 1991 Gastrin‐histamine sequence in the regulation of gastric acid secretion. Gut 32:698–701. 1711995 10.1136/gut.32.6.698 PMC1378893

[phy212793-bib-0047] Wisen, O. , S. Rossner , and C. Johansson . 1987 Gastric secretion in massive obesity. Evidence for abnormal response to vagal stimulation. Dig. Dis. Sci. 32:968–972. 3304891 10.1007/BF01297185

[phy212793-bib-0048] World Health Organization . 2009 Global health risks: mortality and burden of disease attributable to selected major risks. World Health Organization, Geneva.

[phy212793-bib-0049] Yang, Q. , T. E. Graham , N. Mody , F. Preitner , O. D. Peroni , J. M. Zabolotny , et al. 2005 Serum retinol binding protein 4 contributes to insulin resistance in obesity and type 2 diabetes. Nature 436:356–362. 16034410 10.1038/nature03711

[phy212793-bib-0050] Yoshikawa, I. , M. Nagato , M. Yamasaki , K. Kume , and M. Otsuki . 2009 Long‐term treatment with proton pump inhibitor is associated with undesired weight gain. World J. Gastroenterol. 15:4794–4798. 19824113 10.3748/wjg.15.4794 PMC2761557

[phy212793-bib-0051] Zemany, L. , S. Bhanot , O. D. Peroni , S. F. Murray , P. M. Moraes‐Vieira , A. Castoldi , et al. 2015 Transthyretin antisense oligonucleotides lower circulating RBP4 levels and improve insulin sensitivity in obese mice. Diabetes 64:1603–1614. 25524914 10.2337/db14-0970 PMC4407860

[phy212793-bib-0052] Zwirska‐Korczala, K. , S. J. Konturek , M. Sodowski , M. Wylezol , D. Kuka , P. Sowa , et al. 2007 Basal and postprandial plasma levels of PYY, ghrelin, cholecystokinin, gastrin and insulin in women with moderate and morbid obesity and metabolic syndrome. J. Physiol. Pharmacol. 58(Suppl. 1):13–35. 17443025

